# Primi-Dentures

**Published:** 1919-02

**Authors:** F. W. Frahm


### PRIMI-DENTURES

BY F. W. FRAHM, D.D.S.

Temporary Denture—"This is a term frequently applied to dentures inserted immediately upon, or soon after the extraction of the natural teeth, before the resorption of the alveolar process is complete."—*American Text Book of Prosthetic Dentistry.*

The foregoing definition of a denture usually spoken of as a "temporary denture" is as satisfactory as any. But it does not convey to the mind any logical reason why the prosthetic restoration is temporary. Prothero in speaking of "temporary dentures" assumes that the reader is aware of the fact that these exist and have a place in dentistry as such. Turner calls attention to the temporary character of all dentures; that there really is no permanent denture in the full sense of the word. In the real sense of the word "temporary" he is quite right. Everything that man makes is temporary and subject to change and retrogression, but within certain reasonable bounds we may call some of our work permanent, depending upon the purposes we wish to attain.

I would like to define the so-called "temporary denture" as a primi-denture that is to serve a temporary, but important purpose and function.

A large percentage of patients seem to have a well-established idea that these temporary dentures are inferior in every way and not worthy of a reasonable fee or of any care on their part. This unfortunate conception on the part of the patient may be partly due to some ill-advised statement made by some members of the profession, or it may be the outgrowth of mutual assumptions on the part of both. Nevertheless, we are frequently brought face to face with that situation and will have to accept the old version of the "temporary denture" and its small fee, or we will have to educate the patient to the real conditions and needs of the case. We nust show him that the service and skill, required to restore and maintain the best results at this time, should be of the highest type. Let me ask, when does a "temporary denture" cease to exist as such, and become a permanent denture? I can recall with regret many cases for which I made the first denture after extraction had taken place, called it "temporary" and accepted a small fee. I extracted some teeth for my mother late in February, 1905, and made her primi-dentures before April of the same year. While East this summer (1918) I visited at home and learned that mother was eating corn off the cob with these same "temporary dentures." True, they have needed some repairs, but she has never had them refitted over a cast made from a later impression. I am indeed very glad that my mother is receiving the service that these dentures are giving her, but I want to make the point clear, that we can not tell beforehand whether the dentures will be temporary or permanent. Why, then, should we name them temporary and accept a smaller fee? It is not my intention at this time to enter into an economic discussion or consideration of this phase of our work, but to deal with a more important and hitherto untouched phase of denture work that is closely associated with the primi-denture.

After extraction, the question of immediate or delayed prosthesis will always force itself upon the operator and patient. The solution of this question is frequently not based upon physiological reason. The patient is usually consulted regarding the amount of money he or she would be willing to invest without considering the real needs of the case, and not infrequently the operator will advise delaying restoration. He has two reasons for doing so: First, the substitution of the artificial teeth will be easier six to nine months after extraction than if the work is done at once; second, if the restoration is delayed the patient will save one fee, but on the other hand will remain toothless for this period of time. The patients are usually willing to do this because of the saving of one fee and the operator's statement that the case will fit better after resorption is complete.

Fortunately, these two reasons are insignificant when compared with the real and major reason for immediate prosthesis. In looking over the writings of leading prosthetists, I noted the following minor reasons for immediate prosthesis: To serve the patient by giving him teeth upon which to masticate during the interim between extraction and completed resorption; that the patient will more easily adapt himself to the presence and use of the substitute if he does not have to wait months before it is placed in position; that the patient will not have to appear toothless in public and thus avoid the humiliation and unkindly comment in reference to the patient's age, and lastly, that the alveolar ridges will resorb more uniformly under the dentures with a better and firmer ridge upon which to build the permanent dentures as the result.

I shall not comment upon any of the above reasons unless it be the last one. It has been my experience, in replacing a poorly constructed primi-denture, that the ridges were everything but uniform and smooth, and not infrequently such a case becomes a real problem, owing to the careless adaption of a ''temporary denture.''

How soon after extraction shall the substitute for the natural teeth be fitted to the edentulous borders?

At once. Without question. There is no real and sufficient reason for waiting even a day. There may arise some unlooked for complication during and immediately following extraction that would call for a delay of several days. If the impression is not made at once and there should be a swelling of the tissues, I would advise a delay only sufficiently long to allow the inflammation to subside, but not longer, for there is a real and all-important reason why the natural teeth should be replaced with as little delay as possible, especially in those cases where all the teeth have been removed from either or both arches.

As a general rule, the complete substitution of all of the teeth in either or both arches does not take place until after middle life. Hence, during all these years of mastication there has existed a very definite inter-relationship of the several parts that enter into, and make up the tempora-mandibular articulation, and of the articulating surfaces and functions of the teeth. Then after the loss of the natural teeth some men will rush in and make substitutes for these lost organs without regard to the anatomical requirements of the case. Afterwards they defend themselves by telling the patient that an artificial "bite" for these artificial teeth will have to be cultivated. These men seem to be wholly indifferent as to what the ultimate results will be. Truly it has been said, "Men rush in where angels fear to tread." In the past I have been just as guilty of doing this as any man practicing this day. But it was at a time when this branch of dentistry was given very little thought, and although I have made prosthesis a subject of special study for the past fourteen years, I am today more convinced than ever before that it is the most difficult and least understood branch of dental practice; that there are no short cuts to success in this field any more than in any other; that there is no "cure-all" that will bring about perfect success in every case, and that when we do not consider every case as a unit and give it the necessary attention its technique demands, we are not living up to the present-day light that we have on the subject.

A long wait after extraction without a support that will maintain the former anatomical relationship, or the use of an ill-constructed or poorly-adapted substitute will cause a pathological and destructive change to take place in the temporo-mandibular joint that can never be compensated for. Either procedure will make future prosthesis for the patient a problem of such a nature that both operator and patient will become discouraged.

The damage that was thus done to the discus-articularis and the destructive process that was inaugurated in the glenoid fossae and condyles by a well-meaning, but misinformed practitioner can never be corrected or even modified. This is the reason why I am appealing to dentists to make immediate and correct individual application of dentures that harmonize with the anatomical requirements of the case, limited only by the present-day light that we have on the subject.

Dr. H. J. Prentiss of the anatomical department of the University of Iowa, in his preliminary report on the temporo-mandibular articulation,* calls attention to certain destructive changes that take place in the discus-articularis or meniscus, which occur after the articulation of the teeth is changed or lost, stating that the change in the form of the meniscus becomes pathological with the loss of the teeth.

During infancy the meniscus of the temporo-mandibular articulation is more or less uniform in thickness. It does not show any signs of thickening at the edges or thinning in the center until after the eruption of the deciduous teeth. Also, with the eruption of the teeth we can observe the lengthening of the rami in harmony with the demands of the new articulation that is being established by the eruption of these teeth. The relationship that is thus established so early between the teeth, the developing rami and the temporo-mandibular articulation will not be changed to any extent during the normal growth and development of the individual unless interfered with by some pathological or extraneous influence. When the permanent teeth begin to erupt and take the places vacated by the deciduous teeth, it will be in perfect harmony with the in-

---

* *Dental Cosmos*, June, 1918.

creasing length of the rami of the mandible and the development of the other structures that enter into the movements of the mandible during mastication. It is hardly necessary to call your attention to the height of the soft tissues surrounding the newly erupted permanent tooth and which assumes a more normal position as development progresses during the following few years. I also wish to call your attention to the fact that the alveolar process is not fully developed in the early life of the individual. That this band of process supporting the teeth, and the body of the mandible compensates for the increasing length of the rami. Thus the primary relationship that was established early in life between the occlusal plane of the deciduous teeth and the temporo-mandibular articulation is not materially changed during the development of the individual and the eruption of the permanent teeth. The general outline and form of the meniscus is not materially changed from the time of the completion of the two deciduous arches until there is a loss of one or more of the permanent teeth. Therefore, if the normal relationship of the several parts that are concerned with the function of mastication, could be maintained throughout the life of the individual, regardless of the nature of the appliances that are used to supply the teeth that are lost from time to time, the patient would experience little or no difficulty in using such substitutes, even if they should be full maxillary or mandibular dentures, or both. On the other hand, if such scientific care has not been used, or the patient is allowed to masticate or ''gum it,'' without any substitutes, or is compelled to get along as best he can with some makeshift, certain destructive changes will take place in the meniscus that will impair or entirely destroy its function. Not only does the meniscus suffer, but also the condyles and glenoid fossae.

Dr. Prentiss calls attention to the destructive changes that follow the loss of the teeth, stating that the pathological changes may become so aggravated that the remaining plate of bone between the condyle and brain case will be practically nil, and the movements of the mandible will be so limited that the condyles will only rock in the glenoid fossae. This certainly is not a pleasing prospect for the prosthetist or the patient. The practitioner who knowingly ignores this fact and continues to make prosthetic appliances as though every human being had been made like so many castings after one pattern, is willfully neglecting to live up to the maximum of knowledge in this field.

It was my privilege the past summer to call on Dr. Prentiss and to hear him discuss the results of his dissections of the temporo-mandibular articulation. Also his opinions regarding these findings and their influence on dentistry. It also was my privilege to make personal examinations of these specimens and carefully note the destructive changes that had taken place in the discus-articularis of the specimens he had prepared. These, and a number of dissections that I have been able to make in our own anatomical laboratory, supplemented by clinical data, have enabled me to come to the following definite conclusions:

First—The prosthetist is placed at a very decided disadvantage when he is asked to make and supply substitutes for the natural teeth a number of months, or a year after the extractions have been performed, unless the normal distance between the mandible and the maxillae has been maintained by some prosthetic appliance, however crude its construction may have been.

Second—That it is very poor practice, if not indeed malpractice, for a prosthetist to make and supply dentures for a case without recognizing the relationship these substitutes must bear to the temporo-mandibular articulation and the muscles that control mastication.

Third—That the variations and degrees of the inclination of the condyle path increase with age. Likewise the physiological ability of the tissues that form the articulation, to adapt themselves to a change in the working conditions, materially decreases as age advances.

Fourth—A denture or a set of dentures may be made irrespective of the physiological requirements of the patient without any apparent trouble; or the operator may entirely ignore the component parts that are so vitally concerned with the natural function of mastication, and have the patient make some use of the appliance, but, the destructive changes that such a procedure sets up in the temporo-mandibular articulation, can never be restored or compensated for. The meniscus does not possess the power to rebuild itself, neither can the condyle or the glenoid fossae reline themselves with a new coat of cartilage. Any destructive changes that have been forced to take place, due to careless and faulty prosthetic procedure, will remain a serious problem for a number of years, if not for the remainder of the patient's life.

Fifth—The prospect of success in prosthetic procedures in edentulous cases decreases at an ever increasing ratio every time the patient attempts to wear or become accustomed to a denture of which the occlusal relationship to the temporo-mandibular articulation is different from the denture preceding it. This is true, notwithstanding the fact that the last denture may have been as accurate and scientific as it could be made. If the second one does not possess the same scientific relations, it will be more or less a failure. The reason for this is, that the meniscus underwent a change to harmonize with the relationship called for by the former denture. Therefore it is advisable to give careful and detailed consideration to all changes contemplated. Also such changes should not be attempted without regard to the amount and direction of stress to which the denture will be subjected, and its relationship to the movable parts of the temporo-mandibular articulation.

Sixth—That it is a very serious matter, to either open or close the articulation (bite) in any prosthetic procedure, and should only be undertaken under extreme necessity. It matters not if it is crown and bridge work or denture construction, the evil effects on the meniscus, due to this practice, are the same. When a patient has lost some teeth without having them replaced at the time and the remaining teeth have been abraded by the added wear, the change in the glenoid fossae developes so gradually that no inconvenience is experienced. This gradual change, even though extending over a period of years, leads to equally harmful results. To restore this modified relationship to its former state, in a few days, will cause both patient and operator much grief, because the physiological change occurring with advancing age has been turned into a pathological or destructive process. To appreciate to a slight degree the effect of such a procedure, it is only necessary to incorporate several dozen bird shot into one's food, and then chew this food, disregarding their presence in it.

I would like to cite two cases in which I changed the relationship (opened the bite) from what it had been for a period of years. One case of bridge work, the other that of full maxillary and mandibular dentures.

The bridge was in a woman about forty years old, with the teeth badly abraded owing to strong muscular activity and some early extractions. The crowns and bridges changed the relationship, by opening the articulation about five millimeters in the incisor region. It took this patient over six months to even begin to feel at ease, to say nothing of mastication. She was a hearty eater and a healthy and active person, and should have had little difficulty in getting used to any change of a physiological character. The denture was in a man past sixty years of age. His natural teeth had been out for about twelve years and he had worn dentures nearly all of this time, of which the maxillary one was fully six millimeters too short. This was done because there had been an excessive amount of resorption, and the fear that the denture would not stay in place during mastication. Thus, the relationship that existed when the natural teeth were in place was disregarded without any consideration to anatomical and physiological requirements. This man has been compelled, as the result of this, to suffer at the altar of ignorance and negligence. I made the attempt to restore the former relationship about eight years ago, and both the patient and the writer have been having a serious time ever since. It has only been within the past year that we have had any hope or prospect of success; at least he now looks quite normal and "eats corn off the cob" with the dentures. The lesson that the writer has gained through these and other cases causes him now to hesitate to change the former relationship of the several parts that enter into the mastication mechanism.

In conclusion, the smug and self-satisfying theory that the prosthodontist, crown and bridge worker and orthodontist can each establish such an articulation or even only occlusion as he may desire for any or all cases, will pass into the realm of myth after a careful study of the case in life, corroborated at the post-mortem table or anatomical laboratories. I fully appreciate that these post-mortem studies will not be easy to obtain, nevertheless there are some cases in which we may obtain clinical data and then verify such data at the post-mortem examination. The records thus obtained would be of the greatest value to the three branches of practice just named. Scientific men dealing with a scientific problem should seek and demand a scientific solution. Prosthetic dentistry is not simply a mechanical art, surrounded with an esthetic halo, but it is in addition to this, the most really scientific branch of dentistry.—*Pacific Dental Gazette.*

